# Draft Genome Sequence of Bovine Mastitis Isolate *Staphylococcus agnetis* CBMRN 20813338

**DOI:** 10.1128/genomeA.00883-14

**Published:** 2014-09-04

**Authors:** Michael J. Calcutt, Mark F. Foecking, Pamela R. Fry, Hsin-Yeh Hsieh, Jeanette Perry, George C. Stewart, Daniel T. Scholl, Serge Messier, John R. Middleton

**Affiliations:** a Department of Veterinary Pathobiology, University of Missouri, Columbia, Missouri, USA; b Department of Veterinary Medicine and Surgery, University of Missouri, Columbia, Missouri, USA; c Bond Life Sciences Center, University of Missouri, Columbia, Missouri, USA; d Department of Veterinary and Biomedical Sciences, South Dakota State University, Brookings, South Dakota, USA; e Department of Pathology and Microbiology, University of Montréal, Montréal, Québec, Canada

## Abstract

Presented here is a draft genome sequence for *Staphylococcus agnetis* CBMRN 20813338, isolated from a lactating dairy cow with subclinical mastitis. The genome is approximately 2,416 kb and has 35.79% G+C content. Analysis of the deduced open reading frame (ORF) set identified candidate virulence attributes in addition to potential molecular targets for species identification.

## GENOME ANNOUNCEMENT

Staphylococci are common etiological agents of clinical and subclinical bovine mastitis (1). The number of staphylococcal taxa discovered to be associated with intramammary infection recently increased following the isolation of a new species, *Staphylococcus agnetis*, from mastitic milk in Finland (2). Genotypic analysis of three housekeeping genes indicated that this species is closely related to *Staphylococcus hyicus*. *S. hyicus* has been frequently isolated in mastitis studies (3), although the identification of *S. agnetis* now raises the possibility that some *S. hyicus* isolates may, in hindsight, have been *S. agnetis*. As only three genes have been sequenced for *S. agnetis*, a draft genome sequence was determined for a North American mastitic isolate to identify colonization factors and provide additional loci for molecular discrimination from *S. hyicus*. Strain CBMRN 20813338, isolated from a dairy cow in the Canadian Bovine Mastitis Research Network cohort of herds, was selected, as it caused a high milk somatic cell count (3,215,000 cells/ml [normal, <200,000 cells/ml]) in the affected mammary quarter.

Total DNA from *S. agnetis* CBMRN 20813338 was subjected to 454 sequencing and *de novo* assembly (Newbler; Roche) at The Genome Institute at Washington University, St. Louis, MO. The resulting 2,415,997-bp genome was encompassed within 45 contigs, with 46× coverage. Autoannotation was performed using PGAP at NCBI, yielding 2,336 open reading frames (ORFs), 63 tRNAs, and a predicted 6 rRNA-coding operons.

Among the staphylococci for which genomic data are available, the assembled contigs of CBMRN 20813338 are most similar in gene content and organization to those of *Staphylococcus chromogenes* (4), consistent with the 16S rRNA-based phylogeny of these two taxa. Among the regions of variation between *S. chromogenes* and *S. agnetis* is an approximately 7-kb locus in *S. agnetis* harboring genes for putative gas vesicle proteins (5). Although gas vesicle structures have not been reported for any *Staphylococcaceae*, a related gene set was recently identified in two *Staphylococcus simulans* genomes (6). PCR analysis revealed the presence of this locus in porcine *S. hyicus* isolates as well (data not shown).

Noteworthy among the predicted exoproteome of the potential virulence factors are two tandemly arranged ORFs with significant similarity to staphylocoagulase (7), an ORF with a significant match to beta toxin (sphingomyelin phosphodiesterase) of *Staphylococcus aureus* (8), three clustered genes encoding putative superantigens, and a gene encoding a putative hyaluronidase. The hyaluronidase gene was unanticipated, as, among the staphylococci, hyaluronidase genes previously were identified in *S. aureus* only (9). No ORFs with similarity to the trypsin-like proteases of the Exh exfoliative toxin family were identified; such toxins are frequently encrypted in *S. hyicus* strains that cause swine exudative epidermitis (10).

Analysis of the mobile genetic elements revealed two putative prophage genomes and 10 distinct insertion sequences that encode transposases belonging to either the IS*200* (89% identity) or IS*1272* (56% identity) families. No clustered regularly interspaced short palindromic repeat (CRISPR)/Cas loci were detected.

This data set is the first draft genome sequence for the species and provides insight into the features that may contribute to successful host colonization and patterns of gene variation among the *Staphylococcaceae*.

### Nucleotide sequence accession number.

This whole-genome shotgun project has been deposited at DDBJ/EMBL/GenBank under the accession no. JPRT01000000.
